# TRAIL protects the immature lung from hyperoxic injury

**DOI:** 10.1038/s41419-022-05072-5

**Published:** 2022-07-15

**Authors:** Tayyab Shahzad, Cho-Ming Chao, Stefan Hadzic, Judith Behnke, Luisa Biebach, Eva Böttcher-Friebertshäuser, Jochen Wilhelm, Anne Hilgendorff, Klaus-Peter Zimmer, Rory E. Morty, Saverio Bellusci, Harald Ehrhardt

**Affiliations:** 1 Department of General Pediatrics and Neonatology, Justus-Liebig-University and Universities of Giessen and Marburg Lung Center (UGMLC), Feulgenstrasse 12, Giessen, Germany; 2 German Lung Research Center (DZL), Giessen, Germany; 3 Department of Internal Medicine II, Universities of Giessen and Marburg Lung Center (UGMLC), Cardio-Pulmonary Institute (CPI), Aulweg 130, Giessen, Germany; 4 University Medical Centre Rostock, Department of Pediatrics, University of Rostock, Ernst-Heydemann-Strasse 8, Rostock, Germany; 5 Division of Neonatology, University Children’s Hospital, Perinatal Center, Ludwig-Maximilians-University, Campus Großhadern, Marchioninistrasse 15, Munich, Germany; 6 Institute of Virology, Philipps University Marburg, Hans-Meerwein-Strasse 2, 35043 Marburg, Germany; 7 Institute of Lung Biology and Disease and Comprehensive Pneumology Center with the CPC-M bioArchive, Helmholtz Zentrum München, Munich, Germany; 8 Department of Lung Development and Remodeling, Max Planck Institute for Heart and Lung Research, Bad Nauheim, Germany

**Keywords:** Experimental models of disease, Respiratory tract diseases

## Abstract

The hyperoxia-induced pro-inflammatory response and tissue damage constitute pivotal steps leading to bronchopulmonary dysplasia (BPD) in the immature lung. The pro-inflammatory cytokines are considered attractive candidates for a directed intervention but the complex interplay between inflammatory and developmental signaling pathways requires a comprehensive evaluation before introduction into clinical trials as studied here for the death inducing ligand TRAIL. At birth and during prolonged exposure to oxygen and mechanical ventilation, levels of TRAIL were lower in tracheal aspirates of preterm infants <29 weeks of gestation which developed moderate/severe BPD. These findings were reproduced in the newborn mouse model of hyperoxic injury. The loss of TRAIL was associated with increased inflammation, apoptosis induction and more pronounced lung structural simplification after hyperoxia exposure for 7 days while activation of NFκB signaling during exposure to hyperoxia was abrogated. Pretreatment with recombinant TRAIL rescued the developmental distortions in precision cut lung slices of both wildtype and TRAIL^−/−^ mice exposed to hyperoxia. Of importance, TRAIL preserved alveolar type II cells, mesenchymal progenitor cells and vascular endothelial cells. In the situation of TRAIL depletion, our data ascribe oxygen toxicity a more injurious impact on structural lung development. These data are not surprising taking into account the diverse functions of TRAIL and its stimulatory effects on NFκB signaling as central driver of survival and development. TRAIL exerts a protective role in the immature lung as observed for the death inducing ligand TNF-α before.

## Introduction

Bronchopulmonary dysplasia (BPD) is characterized by distortion of lung development mainly in the saccular and early alveolar stage leading to life-long restrictions in lung function in former preterm infants [1]. Central to the pathogenesis is the oxygen induced inflammatory response in the lung which is characterized by the release of pro-inflammatory cytokines like interleukin 1β (IL-1β), interleukin 6 (IL-6) and tumor necrosis factor α (TNF-α) and the subsequent influx of inflammatory cells which reinforces the response and executes lung tissue injury [2]. Thus, alleviating the inflammatory response represents a highly attractive approach to prevent BPD as was successfully implemented in many other inflammatory lung diseases including asthma as well chronic adult lung diseases like COPD [3]. For BPD, a more selective therapeutic approach has not been established successfully so far and the number of available drugs with proven efficacy is very limited [4]. Corticosteroids efficiently dampen the inflammatory response but are nowadays reserved for rescue therapy due to their tremendous side effects on further lung development and psychomotor function [5]. The upcoming evidence in preclinical studies on fibroblast growth factor 10 (FGF-10) and platelet-derived growth factor AA (PDGF-AA) and one first phase II clinical trial on insulin-like growth factor 1 (IGF-1) encourage the search for further novel therapies to balance the equilibrium of cytokine and growth factor action [6–10].

The complex signaling network interplay between inflammatory and developmental pathways requires the detailed evaluation of each candidate as many of them impact on central signaling pathways of the lung like transforming growth factor β (TGF-β) and nuclear factor “kappa light chain enhancer” of activated B cells (NFκB) that are concurrently involved in lung development and lung injury [2]. In line, our recent studies on TNF-α revealed that it is both involved in lung development and disease. The more pronounced pathway imbalance between NFκB and TGF-β signaling in the TNF-α knockout animals resulted in an increased inflammatory response and cell death induction in the lung [11]. On the other hand, the loss of Fas ligand (FasL), another key member of the TNF family, attenuated injury to the immature lung [12]. The documented phenotype differences for TNF-α and FasL can be attributed to the diverse functionalities on cell death, survival and proliferation within this cytokine family that display a highly complex and disease specific action [13]. TRAIL constitutes the third important member of the TNF family. It has been recognized as a regulator of immune responses besides its potent apoptosis inducing capacity and its role in lung diseases has been both described beneficial and detrimental [14, 15]. TRAIL exerts a protective function in pulmonary fibrosis and is crucial for the resolution of chronic allergic airway inflammation. But in rodent models, it aggravates cigarette-smoke induced COPD and influenza virus infection where fundamental pathogenicity was ascribed to the release of TRAIL from inflammatory monocytes [14, 16–18]. A recent observational study in asthma patients documented a differentiated function of TRAIL. A positive association of TRAIL levels in the bronchoalveolar lavage was seen with the type 1, 2 and 17, but not the type 9 inflammatory response and TRAIL levels were not correlated with any parameters of airway remodeling [19].

Here, we evaluated the function of TRAIL in BPD development and as a therapeutic target. We combined analyses of TRAIL cytokine levels in tracheal aspirates from ventilated preterm infants and investigations in transgenic animals. Besides morphologic studies on the consequences of exposure to different concentrations of oxygen, the focus was directed towards detecting the discrepancies in the pulmonary inflammatory response between wildtype and TRAIL^−/−^ mice. Finally, the therapeutic potential of recombinant TRAIL to prevent BPD was evaluated in vitro on precision cut lung slices exposed to hyperoxia.

## Materials and methods

For Western Blot, anti- α-SMA (1:1000, 113200) was obtained from Calbiochem (San Diego, CA, USA), anti-p-IκBα (1:1000, 2859) and anti-IκBα (1:1000, 9242) from Cell Signaling Technology (Danvers, MA) and anti-GAPDH (1:5000, MA1-22670) from Thermo Fisher Scientific (Waltham, MA). For immunofluorescence staining, anti-SPC (1:500, proSP-C) was purchased from Merck Millipore (Burlington, MA, USA), anti-ki67 (1:200, SP6) from Novus Biologicals (Littleton, CO, USA), anti-CD31 (1:100, D8V9E), anti-PDGFRα (1:500, D1E1E) and anti-cleaved-Casp-3 (1:200, Asp175) from Cell Signaling, anti-PDGFRα from Santa Cruz (1:50, C-20), anti-F4/80 (1:100, ab6640) and anti-Ly6G (1:50, ab2557) antibody from abcam (Cambridge, UK), all secondary antibodies from Thermo Fisher Scientific. For anti-cleaved-Casp-3 signal stain boost IHC detection reagent (Cell Signaling) was used for improved signal detection. All antibodies used in this manuscript can be found in SciCrunch database. Animal-free recombinant TRAIL (315-19-50UG) was obtained from PeproTech (Hamburg, Germany).

### Newborn mouse model of hyperoxic injury

#### Transgenic animals

TRAIL knockout mice (B6.129-*Tnfsf10*^*tm1Sdg*^) and C57BL/6 wild type control animals were obtained from Amgen (Thousand Oaks, CA) [20]. Speed congenic allelic profiling performed by Charles River Genetic Testing Service (Sulzfeld, Germany) prevailed 99.74% homology between wild type and TRAIL knockout mice and 99.7% homology of wild type animals compared to reference allelic profiles of C57BL/6 N mice.

#### Animal procedures

Newborn mice were exposed to 21%, 40% or 85% of oxygen at equal gender for seven days within the first twelve hours of life. Animals were sacrificed at the end of hyperoxia or after a recovery period of 21 days in room air by intraperitoneal injection of a lethal dose of pentobarbital. Lungs were harvested en bloc for further analyses after intracardial perfusion with PBS at a pressure of 20cmH_2_0 and directly frozen at −80°. For fixation with paraformaldehyde (PFA), lungs were perfused intratracheally after binding with a string with buffered 4% PFA with a pressure of 20 cmH_2_O and placed in 4% PFA for 24 h at 4 °C, then progressively dehydrated (30%, 50%, 70%, 99,6% ethanol) for 3 h each and embedded with a Leica embedding machine (EG 1150 C). Nursing dams were rotated between normoxia and 85% of hyperoxia at 12 h intervals. All scientific procedures on living animals were approved by the regional council (RP Giessen Az: V 54 - 19 c 20 15 h 01 GI20/12 Nr. 04-2016) in accordance with the German animal welfare law and the European legislation for the protection of animals used for scientific purposes (2010/63/EU). The subsequent TRAIL rescue experiments on precision cut lung slices were performed in accordance with the German animal welfare law and had been declared to the Animal Welfare Officer of the University (Registration No.: M_742_M). The methods used to euthanise the animals humanely were consistent with the recommendations of the AVMA Guidelines for the Euthanasia of Animals.

#### Lung histology and immunofluorescence

Paraffin blocks were kept cold and 2 or 5 μm sections were cut from in vivo lung experiments and 3 µm sections from PCLS. After staining with haematoxylin and eosin, mean linear intercept, air space and mean septal wall thickness were analysed from scans from the right upper lobe using a Leica DM6000B microscope with an automated stage and Qwin V3 software (Leica, Wetzlar, Germany) as published before [6, 9, 21]. Bronchi and vessels >50 µm in diameter were excluded from the analysis.

For immunofluorescence staining, paraffin sections were deparaffinized and antigen unmasking was performed by incubation of the slides in boiling 0.93% citrate buffer solution with pH of 6 for 15 min for anti-KI-67, anti-CD31, anti-PDGFRα, anti-cleaved-Casp-3 and anti-Ly6G staining or treatment with proteinase K for 10 min for anti-F4/80 staining. Slides were blocked with 3% bovine serum albumin (BSA) and 0.4% Triton X-100 [in Tris-buffered saline (TBS)] at room temperature (RT) for 1 hour and then incubated with primary antibodies at 4 °C overnight. After incubation with primary antibodies, slides were washed three times in TBST for 5 min, incubated with secondary antibodies at RT for 1 h, and then washed three times in TBST before being mounted with Prolong Gold Anti-fade Reagent with DAPI (Thermo Fisher Scientific). TUNEL staining (manufacturer Promega) was performed according to the manufacturer´s instructions. Fluorescent images were acquired using Leica DM5500 B fluorescence microscope connected to Leica DFC360 FX camera (Leica, Wetzlar, Germany). Quantification was executed with immunofluorescence positive cells set against the number of DAPI stained nuclei of at least eight frames with a minimum of 250 cells for each biological replicate.

#### Precision cut lung slices (PCLS)

Newborn mice were sacrificed on P1, and the pulmonary vasculature was perfused with sodium chloride 0.9% solution after incision of the left ventricle under sterile conditions at 20 cm H_2_0 to remove hematopoietic cells. After tracheal incision, lungs were filled with pre-heated 3% low-melting agarose before tracheal ligation and embedding in 2% agarose blocks. Lung sections were cut at 150 µm using Microm HM 650 V (Thermo Fisher Scientific). Precision cut lung slices (PCLS) were immediately put in DMEM medium (Thermo Fisher Scientific) supplemented with 10% fetal calf serum. After cell culture adaptation overnight, PCLS were exposed to 21% or 80% of oxygen for 3 days. After washing with PBS, PCLS were deep frozen for western blot or fixed with 4% paraformaldehyde (Santa Cruz Biotechnology), paraffin embedded and further processed as described for the lung tissue before.

#### Real-time PCR

RNA was isolated exclusively from the total upper left lobe using the RNeasy Mini Kit (Qiagen GmbH, Hilden, Germany). RNA was reverse-transcribed using QuantiTect Reverse Transcription Kit (Qiagen GmbH). cDNA was diluted to a concentration of 5 ng/μl. Sybr Green Master Mix (Invitrogen, Carlsbad, CA) was used for RT-PCR with a Roche LightCycler 480 machine. Samples were run in triplicates for IL-1β, TNF-α, and TGF-β1 using Hprt as reference gene. Primers are listed in Supplementary Table 1.

#### Western blot analysis

Lung tissue was lysed in total cell lysis buffer RIPA (Santa Cruz). 5% SDS in bromophenol blue and b-mercaptoethanol loading buffer was added to protein samples, denatured for 5 min at 95 °C and cooled on ice. 5–30 μg of protein was loaded on 8–15% polyacrylamide gels, then transferred to a polyvinylidene fluoride (PVDF) membrane (Amersham, Germany) by semi-dry electro blotting. After blocking with 5% milk in TBS-T at room temperature on a shaker for 1 h, incubation with the primary antibody was conducted overnight at 4 °C. After washing with TBS-T three times for 10 min, the membrane was incubated with the respective HRP-labelled secondary antibody at room temperature for 1 h followed by three times washing with 1 X TBS-T buffer for 10 min. Chemiluminescence signals were detected with SuperSignal West Femto kit (Thermo Fisher Scientific). An internal standard on each gel enabled the comparison between different gels. Bands quantification was performed using ImageJ software version 1.8.0_112.

### TRAIL levels in tracheal aspirates of preterm infants

Tracheal aspirates were obtained from preterm infants (*n* = 69) who failed successful stabilization on non-invasive ventilation at birth. Of these, *n* = 32 required prolonged invasive mechanical ventilation with oxygen rich gas for at least 14 days and were available for repeated measurement. Levels of TRAIL were determined by a commercial kit (R&D Systems, Minneapolis, MN) standardized to sIgA (Immundiagnostik AG, Bensheim, Germany) as described [8]. The studies were approved by the ethics committee of the Ludwig-Maximilians-University Munich (no. 195-07) and of the Justus-Liebig-University Gießen (no. 135-12) in accordance with the declaration of Helsinki and written parental consent was obtained in all cases. The study was registered at the German Clinical Trials Register (DRKS00004600).

### Statistical analysis

Data are given as mean and SEM. The required sample size of *n* = 6 for each experimental setting was calculated using GPower version 3.1. at a significance level of *p* = 0.05 with an estimated effect size of 0.85 and a power > 0.8. Student’s *t*-test or rank sum test when normality test failed were used to test for statistically significant differences. Statistical analyses were performed using Sigma Plot 12.3. (Systat Software, San Jose, CA). Differences were considered significant at *p*-values < 0.05.

## Results

### Reduced levels of TRAIL in tracheal aspirates of infants with BPD

**Fig. 1:**
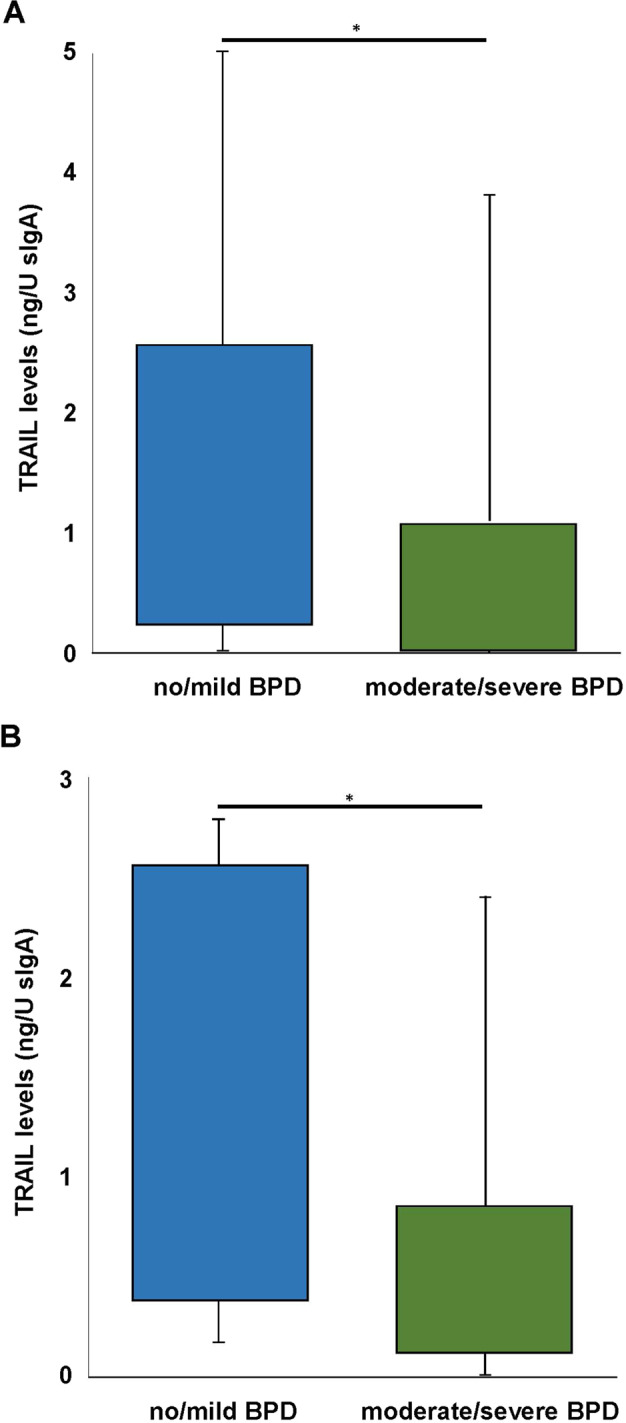
Lower TRAIL levels in tracheal aspirates of preterm infants associated with moderate/severe BPD. **A** Tracheal aspirates of *n* = 33 preterm infants with no/ mild BPD and of *n* = 36 with moderate/severe BPD obtained directly after birth were analyzed for TRAIL standardized to sIgA. Levels of TRAIL were significantly reduced in infants developing moderate/severe BPD. **B** Of these infants from **A**, *n* = 8 infants with no/ mild BPD and *n* = 24 with moderate/severe BPD required prolonged mechanical ventilation until day 14 of life. Tracheal aspirates were analyzed as in **A**. TRAIL levels persisted significantly reduced in infants developing moderate/severe BPD. Data are presented as median, interquartile range and complete range. Statistical analysis was performed by rank-sum test. *p < 0.05.

To dissect the role of TRAIL in the pathogenesis of BPD in the preterm infant we performed an observational study in a prospective cohort of preterm infants (<29 weeks of gestation), and determined the levels of TRAIL in tracheal aspirates of *n* = 69 infants which required intubation and mechanical ventilation at birth with the expected predominance of male gender (*n* = 45). *n* = 32 infants still depended on mechanical ventilation with oxygen rich gas after 14 days of which *n* = 21 were of male gender. Sex ratios did not differ between the no/mild and the moderate/severe BPD comparison groups. Congruent with our previous results for TNF-α [11], TRAIL levels were significantly higher in tracheal aspirates of preterm infants at birth who did not develop moderate or severe BPD (Fig. 1A). This significant difference persisted in the subset of infants that depended on prolonged mechanical ventilation for at least 14 days (Fig. 1B). These results formed the basis for the comprehensive evaluation of TRAIL in the pathogenesis of BPD within the newborn mouse model.

### Increased lung injury in TRAIL^−/−^ mice after exposure to 40% and 85% of oxygen

**Fig. 2:**
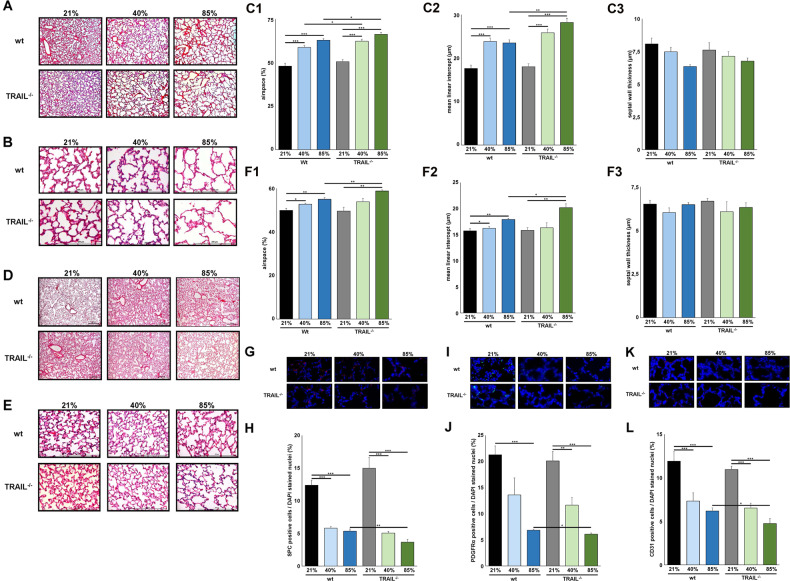
Loss of TRAIL aggravates hyperoxia-induced alveolar simplification in newborn mice. **A** Haematoxylin/eosin staining of representative lung tissue sections from newborn wildtype and TRAIL^−/−^ mice exposed to 21%, 40% or 85% of oxygen for 7 days starting after birth. Hyperoxia induced alveolar simplification was visible at 40% and 85% of oxygen. Scale bar: 200 µm. **B** Enlarged image sections from haematoxylin/eosin stainings as in **A**. Scale bar: 200 µm. **C** Corresponding lung morphometric analyses for airspace (left panel), mean linear intercept (middle) and septal wall thickness (right panel) from **A**. Hyperoxia-induced increase in airspace was significantly more pronounced in TRAIL^−/−^ compared to wildtype mice for 40% and 85% of oxygen. For mean linear intercept, statistical significance was reached at 85% of oxygen. **D** Mice from **A** were allowed to recover for another 21 days in room air after exposure to hyperoxic injury before histologic evaluation. Hyperoxia-induced alveolar simplification persisted for 40% and 85% of oxygen. Scale bar: 200 µm. **E** Enlarged image sections from haematoxylin/eosin stainings as in **D**. Scale bar: 200 µm. **F** Lung morphometric analyses for airspace (left panel), mean linear intercept (middle) and septal wall thickness (right panel) were executed for haematoxylin/eosin stainings from **D**. The statistically significant increase in airspace and mean linear intercept after hyperoxic injury in TRAIL^−/−^ compared to wildtype mice persisted for 85% of oxygen after the recovery period of 21 days in room air. **G** Immunofluorescence staining for surfactant protein C (SPC) positive cells of lung tissues from mice from **A**. Hyperoxia-induced rarefication of SPC cells was detectable at 40% and 85% of oxygen. **H** Quantification of SPC positive cells from **G**. Hyperoxia-induced rarefication of SPC positive cells was significantly more pronounced in TRAIL^−/−^ compared to wildtype mice for 85% of oxygen. **I** Immunofluorescence staining for platelet derived growth factor receptor α (PDGFRα) positive cells of lung tissues from mice from **A**. Hyperoxia-induced rarefication of PDGFRα cells was visible at 40% and 85% of oxygen. **J** Quantification of PDGFRα positive cells from **I**. Hyperoxia-induced rarefication of PDGFRα positive cells was significantly more pronounced in TRAIL^−/−^ compared to wildtype mice for 85% of oxygen. **K** Immunofluorescence staining for vascular endothelial CD31 positive cells of lung tissues from mice from **A**. Hyperoxia-induced rarefication of CD31 cells was apparent at 40% and 85% of oxygen. **L** Quantification of CD31 positive cells from **K**. Hyperoxia-induced rarefication of CD31 positive cells was significantly more pronounced in TRAIL^−/−^ compared to wildtype mice for 85% of oxygen. Data are presented as mean + SEM. Statistical analysis was performed by *t*-test. *p < 0.05, **p < 0.01, ***p < 0.001, ****p < 0.0001. *n* ≥ 6 mice/group.

To test the consequences of hyperoxia exposure, wildtype and TRAIL^−/−^ mice were exposed to 40% or 85% of oxygen for the first 7 days of life mimicking the clinical situation of moderately and severely lung compromised preterm infants. Survival rates until P8 were reduced in animals exposed to 85% of hyperoxia but did not differ significantly between wildtype (89.1%) and TRAIL^−/−^ (87.3%) animals. As well, weight gain was similar between the respective groups of wildtype and TRAIL^−/−^ animals. For exposure to 85% of oxygen, mean weight was 3.641 (SEM 0.0933, *n* = 41) grams in wildtype and 3.417 (SEM 0.135, *n* = 35) grams in TRAIL^−/−^ animals (*p* = 0.177) that was not significantly different from normoxic controls. Both in wildtype and transgenic animals, alveolar sacs were more enlarged and mean linear intercept measurements were higher for 85% than for 40% of oxygen exposure (Fig. 2A, B). Standardized morphometric analyses revealed significantly increased airspaces in TRAIL^−/−^ compared to wildtype animals for both 40% and 85% of oxygen exposure. Airspace and mean linear intercept were significantly higher in TRAIL^−/−^ than in control mice at 85% of oxygen, while septal wall thickness was not affected (Fig. 2C). These structural alterations persisted when mice were followed up after a recovery period of 21 days in room air (Fig. 2D–F).

Separate investigation of the different lung cell populations after 7 days of hyperoxic injury revealed that 40% and 85% of oxygen significantly reduced the number of surfactant protein C (SPC) positive type II cells, platelet-derived growth factor receptor α (PDGFRα) positive mesenchymal progenitors and CD31 positive vascular endothelial cells both in wildtype and TRAIL^−/−^ mice (Fig. 2G–L). Semiquantitative analyses showed a significant reduction of all three cell populations in TRAIL^−/−^ compared to wildtype mice exposed to 85% of oxygen (Fig. 2H–L).

The data obtained so far in biosamples from preterm infants and newborn mice allow the conclusion that TRAIL exerts a protective effect for structural development in the immature lung exposed to hyperoxia. To further dissect the pathomechanisms, we directed the focus towards the injury patterns in wildtype and TRAIL^−/−^ mice.

### Increased inflammation and apoptosis induction in TRAIL^−/−^ mice exposed to hyperoxia

**Fig. 3:**
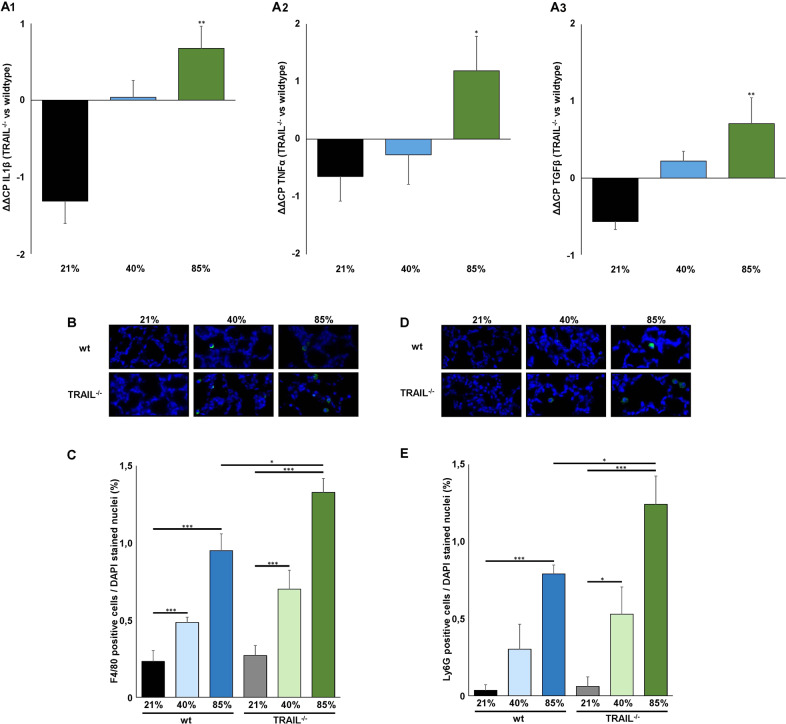
Increased inflammation in lungs of TRAIL^−/−^ compared to wildtype mice exposed to hyperoxia. **A** Real-time qPCR for IL-1β (upper panel), TNF-α (middle) and TGF-β (lower panel) in lungs of newborn wildtype and TRAIL^−/−^ mice. Data from mice exposed to 21%, 40%, or 85% of oxygen for 7 days are presented as ΔΔCP values = (ΔCP TRAIL^−/−^) – (ΔCP wildtype), with ΔCP = (CT reference gene) – (CT gene of interest). Hyperoxia-induced upregulation of IL-1β, TNF-α and TGF-β was significantly increased in TRAIL^−/−^ compared to wildtype mice at 85% of oxygen. **B** Immunofluorescence staining for F4/80 positive macrophages of lung tissues from mice from Fig. 2A. Hyperoxia-mediated influx of macrophages into the lungs of newborn mice was detectable at 40% and 85% of oxygen. **C** Quantification of F4/80 positive macrophages from **B**. Hyperoxia mediated influx of macrophages was significantly increased in TRAIL^−/−^ compared to wildtype animals at 85% of oxygen. **D** Immunofluorescence staining for Ly6G positive neutrophils of lung tissues from mice from Fig. 2A. Hyperoxia mediated influx of neutrophils into the lungs of newborn mice was demonstrated at 40% and 85% of oxygen. **E** Quantification of Ly6G positive neutrophils from **D**. Hyperoxia mediated influx of neutrophils was significantly increased in TRAIL^−/−^ compared to wildtype animals at 85% of oxygen. Data are presented as mean + SEM. Statistical analysis was performed by *t*-test. *p < 0.05, **p < 0.01, ***p < 0.001, ****p < 0.0001. *n* ≥ 6 mice/group.

**Fig. 4:**
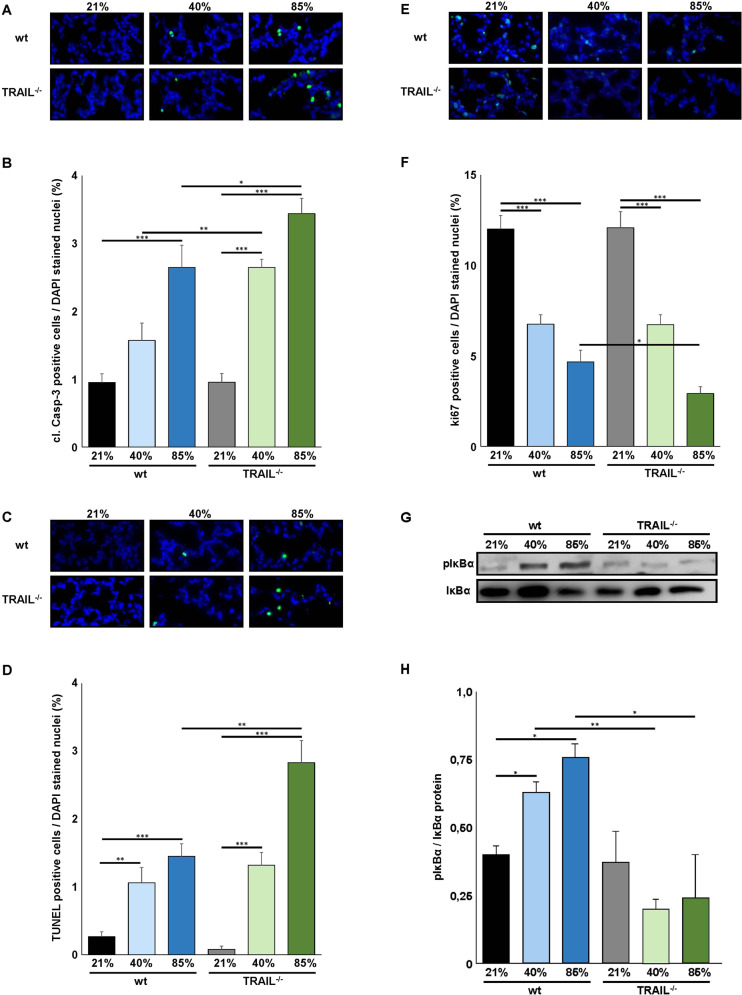
Increased apoptosis induction and reduced proliferation in lungs of TRAIL^−/−^ compared to wildtype mice exposed to hyperoxia. **A** Immunofluorescence staining for cleaved Caspase-3 (cl-Casp-3) positive cells of lung tissues from mice from Fig. 2A. Cleavage of Caspase-3 was detected in the lungs of newborn mice exposed to 40% and 85% of oxygen. **B** Quantification of cl-Casp-3 positive cells from **A**. Hyperoxia-induced cleavage of Caspase-3 was significantly increased in TRAIL^−/−^ compared to wildtype animals at 85% of oxygen. **C** Immunofluorescence staining for TUNEL positive cells of lung tissues from mice from Fig. 2A. Hyperoxia-induced apoptosis induction in the lungs of newborn mice was apparent at 40% and 85% of oxygen. **D** Quantification of TUNEL positive cells from **C**. Hyperoxia-mediated apoptosis induction was significantly increased in TRAIL^−/−^ compared to wildtype animals at 85% of oxygen. **E** Immunofluorescence staining for ki67 positive cells of lung tissues from mice from Fig. 2A. Hyperoxia-induced reduction of ki67 positive cells was present at 40% and 85% of oxygen. **F** Quantification of ki67 positive cells from **E**. Hyperoxia-induced reduction of ki67 positive cells was significantly increased in TRAIL^−/−^ compared to wildtype animals at 85% of oxygen. **G** Western blot analysis of p-IκBα and IκBα from lungs from mice from Fig. 2A. Wildtype and TRAIL^−/−^ mice displayed equivalent phosphorylation of IκBα at 21% of oxygen. p-IκBα levels increased only in wildtype mice exposed to hyperoxia. **H** Quantification of p-IκBα from **G**. Levels of p-IκBα remained unchanged in TRAIL^−/−^ mice while phosphorylation of IκBα significantly increased in wildtype animals at 40% and 85% of oxygen. Data are presented as mean + SEM. Statistical analysis was performed by *t*-test. *p < 0.05, **p < 0.01, ***p < 0.001, ****p < 0.0001. *n* ≥ 6 mice/group.

To identify the disparities of pathophysiologic alterations in the lungs of wildtype and TRAIL^−/−^ animals, the subsequent analyses were directed towards inflammation and apoptosis induction. As expected, 40% and 85% of oxygen both induced a pronounced inflammatory response. The classical pro-inflammatory cytokines IL-1β, TNF-α and TGF-β were more intensely upregulated in lung tissues from TRAIL^−/−^ compared to wildtype mice exposed to oxygen that reached statistical significance at 85%. This increase in cytokine inflammation was accompanied by a significantly augmented influx of macrophages and neutrophils (Fig. 3A–E). The more pronounced inflammatory response in the lungs went along with an increased apoptosis induction. The extent of Caspase-3 cleavage was significantly increased in TRAIL^−/−^ animals exposed to 40% and 85% of oxygen. In line, the number of TUNEL positive cells was higher in TRAIL^−/−^ exposed to 85% of oxygen (Fig. 4A–D). Vice versa, cell proliferation measured by ki67 staining prevailed significantly more attenuated in TRAIL^−/−^ mice exposed to 85% of oxygen. This went along with a reduced activation level of the classical NFκB pathway and phosphorylation of IκBα in TRAIL^−/−^ mice exposed to 40% and 85% of oxygen (Fig. 4E–H).

Taken together, the loss of TRAIL aggravates lung inflammation and injury in the immature lung exposed to hyperoxia. Pathologic changes were comparable to that observed for TNF-α before [11].

### Pretreatment with TRAIL abrogates the hyperoxia-induced structural changes in PCLS of wildtype and TRAIL^−/−^ mice

**Fig. 5:**
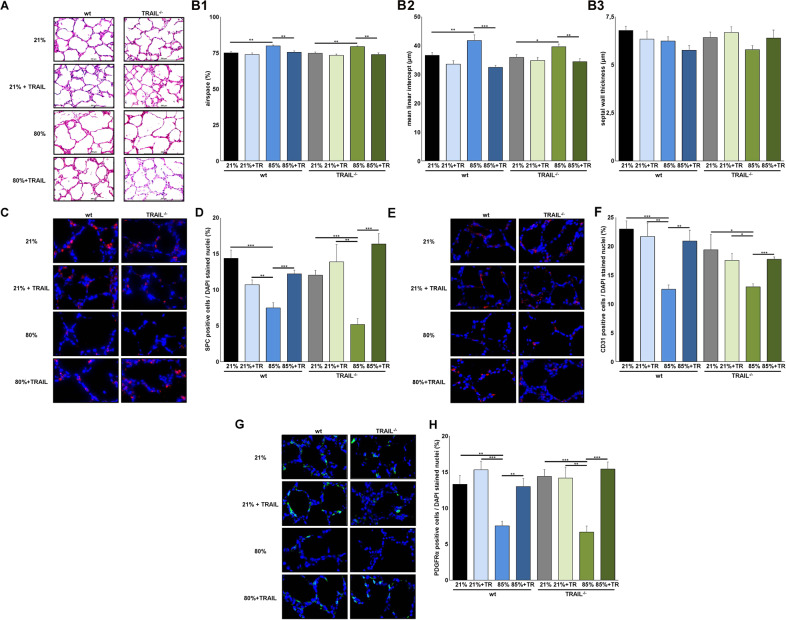
Pretreatment with recombinant TRAIL attenuates hyperoxia-induced lung injury in PCLS from wildtype and TRAIL^−/−^ mice. **A** Haematoxylin/eosin staining of precision cut lung slices (PCLS) of newborn mice pretreated with recombinant TRAIL (100 ng/ml) at P2 before exposure to 21% or 80% of oxygen for 3 days. PCLS were re-stimulated with TRAIL every 24 h. PCLS displayed the characteristic morphologic features of hyperoxic injury after 72 h similar to the in vivo situation presented in Fig. 2A–C that was inhibited by pretreatment with TRAIL. Scale bar: 200 µm. **B** Corresponding lung morphometric analyses for airspace (left panel), mean septal wall thickness (right panel) and linear intercept (lower panel) of PCLS from **A**. Hyperoxia-induced increase in airspace and mean linear intercept was present to an equal extent in PCLS from wildtype and TRAIL^−/−^ mice. TRAIL pretreatment rescued lung development both in wildtype and TRAIL^−/−^ animals. **C** Immunofluorescence staining for SPC positive alveolar type II cells in PCLS from **A**. PCLS from wildtype and TRAIL^−/−^ animals exhibited rarefication of SPC positive cells after hyperoxic injury that was inhibited by pretreatment with TRAIL. **D** Quantification of SPC positive cells from **C**. Hyperoxia-induced rarefication of SPC positive cells was similar in PCLS from wildtype and TRAIL^−/−^ mice. TRAIL pretreatment rescued the presence of SPC positive cells both in wildtype and TRAIL^−/−^ animals. **E** Immunofluorescence staining for CD31 positive vascular endothelial cells in PCLS from **A**. PCLS from wildtype and TRAIL^−/−^ animals demonstrated rarefication of CD31 positive cells after hyperoxic injury that was inhibited by pretreatment with TRAIL. **F** Quantification of CD31 positive cells from **E**. Hyperoxia-induced rarefication of CD31 positive cells was similarly pronounced in PCLS from wildtype and TRAIL^−/−^ mice. TRAIL pretreatment rescued the presence of CD31 positive cells both in wildtype and TRAIL^−/−^ animals. **G** Immunofluorescence staining for PDGFRα positive mesenchymal progenitor cells in PCLS from **A**. PCLS from wildtype and TRAIL^−/−^ animals displayed rarefication of PDGFRα positive cells after hyperoxic injury that was inhibited by pretreatment with TRAIL. **H** Quantification of PDGFRα positive cells from **G**. Hyperoxia-induced rarefication of PDGFRα positive cells was similarly pronounced in PCLS from wildtype and TRAIL^−/−^ mice. TRAIL pretreatment rescued the presence of PDGFRα positive cells both in wildtype and TRAIL^−/−^ animals. Data are presented as mean + SEM. Statistical analysis was performed by *t*-test. *p < 0.05, **p < 0.01, ***p < 0.001, ****p < 0.0001. *n* ≥ 3 mice/group.

To further verify the function of TRAIL for lung development, we performed TRAIL rescue experiments in PCLS that were depleted of leucocytes and therefore of the hematologic inflammatory response to hyperoxia. PCLS displayed the identical structural simplifications characteristic for oxygen toxicity with enlarged airspaces and increased mean linear intercepts when exposed to 80% of oxygen for 72 h. These alterations were equally pronounced in PCLS from wild-type and TRAIL^−/−^ mice (Fig. 5A, B). Pretreatment and repetitive application of recombinant TRAIL during hyperoxia led to the preservation of structural lung development under 80% of oxygen both in wild type and TRAIL^−/−^ PCLS (Fig. 5A, B). The investigation of SPC positive alveolar type II cells revealed a dramatic reduction in PCLS from wild type and TRAIL^−/−^ mice that was prohibited by recombinant TRAIL. Similarly, the decrease of CD31 positive vascular endothelial cells and of PDGFRα positive mesenchymal progenitors was averted to a similar extent in wildtype and TRAIL^−/−^ PCLS (Fig. 5C–H).

These data provide further evidence that TRAIL can alleviate the pathologic changes induced by hyperoxia in the immature lung.

## Discussion

All published data on targeting the imbalance of pro-inflammatory cytokines and stunted growth factor signaling in the immature lung disclosed that this represents a highly attractive approach to prevent BPD [22, 23]. But the complex interplay between lung growth promoting and injurious signaling pathways make the precise dissection of cytokine and growth factor signaling in lung development and disease mandatory [2]. With respect to novel interventions, the so far most promising data are available for growth factor substitution in a situation of deficiency including FGF-10, PDGF-AA and IGF-1 but results are mainly restricted to preclinical trials [2, 6–10]. Vice versa, the overweight of pro-inflammatory cytokines with their detrimental action encouraged studies to inhibit their activity. But results for the pro-inflammatory cytokine TNF-α argue towards caution to completely block its action as baseline activity seems to be required for proper lung development in the saccular stage [11]. Our data on TRAIL add another cytokine to the list that exerts lung growth promoting activity under physiologic conditions. The clinical together with the experimental data on a persistent beneficial effect during the lung injury and a recovery period argue towards categorization of TRAIL distinct from TNF-α where excess activation was associated with more severe lung injury and BPD [24, 25].

The PCLS technique allowed further insights into the role of TRAIL during lung injury. While in vivo, the loss of TRAIL was accompanied by an increased inflammatory response in the lung during hyperoxia and aggravated lung injury, the extent of lung damage by hyperoxia was equivalent in PCLS from wildtype and TRAIL^−/−^ mice when hematopoietic cells were depleted. The data allow the specification of hyperoxia induced lung injury and the additional damage by excess inflammation that was observed in TRAIL^−/−^ mice. Although the direct toxic effect of hyperoxia was the predominant injurious factor, disparities in inflammation account for clinically relevant variations [26]. These data confirm that targeting the inflammatory response during injury of the immature lung prevails a research priority that improves the pulmonary outcome. As described in TNF-α^−/−^ mice before [11], the aggravated injury can be ascribed to the impaired activation and survival function of NFκB signaling while TGFβ excess can aggravate lung injury [27, 28]. While TNF-α and TRAIL induce their pro-survival and pro-proliferative action via different cell surface receptors, these converge on common signaling pathways [13, 29, 30].

Therefore, restitution of physiologic TRAIL levels during injury seems promising based on the clinical cohort results and the preclinical in vivo data presented here. Within the influenza virus lung injury model, macrophages were described as a major source of TRAIL [16]. It will be important to determine disparities in TRAIL release from these cells in infants with better and worse pulmonary outcome within future biosample studies. To further study the utility of recombinant TRAIL application, we established the novel treatment model with PCLS from newborn mice. Therapeutic application prevailed beneficial both in PCLS of wildtype and TRAIL depleted animals. The obtained results even argue towards even advanced lung growth in normoxia and hyperoxia.

Our data provide novel mechanistic insights on the role of the prominent members of the TNF superfamily in the pathogenesis of BPD. While targeting the death inducing ligand FasL prevailed beneficial, the identical approach to TNF-α aggravated lung injury and depleted mesenchymal progenitors [11, 12]. Our data on TRAIL rise further complexity to the topic within one cytokine family as TRAIL protects the immature lung even during prolonged hyperoxic exposure in vitro and in vivo. Of importance, its action is directed towards several key cell populations of the three compartments of the lung. This utility is mediated via NFκB in the absence of any observed adverse effects on lung development and is in line with the recent observations for TNF-α. Within the clinical cohort, the most severely affected infants with moderate and severe BPD displayed significantly lower levels of TRAIL in their lungs. These infants have the highest risk for severe alveolar simplification and reduction in surface area for gas exchange which is accompanied by rarefication of the pulmonary vasculature [1, 31]. These pathologies put them at special risk for severe lifelong morbidities and death. Our data suggest the inclusion of TRAIL as a marker of disease severity and for treatment monitoring into future clinical trials.

The observed function of TRAIL is surprising at first sight as TRAIL is mainly attributed for its pro-apoptotic function and was originally studied as a cancer therapeutic in this context [32]. But data from the last two decades of research established its alternative pro-survival and pro-proliferative function during development and tissue homeostasis [33–36]. In line, TRAIL proved beneficial or detrimental in different pulmonary diseases in a strict context specific manner [14, 16–19]. These disparate functions can be retrieved to the divergent intracellular signaling via the TRAIL receptors which displays a context and situation specific action [27]. This decision between life and death favors in our studies the activation of the classical pathway of NFκB in the immature lung which is well-known for its pro-survival and pro-proliferative action [27]. The abrogation of NFκB activation in TRAIL^−/−^ mice exposed to hyperoxia has probably enabled the aggravation of lung injury in our experiments induced by factors like TGFβ [11, 28]. This has been detailed within inhibition studies of NFκB during hyperoxic or infectious injury before that prevailed a detrimental effect to the immature lung [27, 37, 38]. Further elaboration of the function of NFκB requires lineage-specific deletion models and deletion models of downstream targets of NFκB.

Not surprisingly, the application of recombinant TRAIL efficiently protected PCLS from the key characteristics of BPD induced by hyperoxia, the stunted alveolar and vascular development. All compartments of the lung benefited from its presence in vivo and its therapeutic application in vitro. The data for TRAIL are not surprising taking into account the previous publications on the relevance of NFκB signaling to attenuate lung injury [27]. Our data underpin the need for therapies that preserve physiologic NFκB signaling in the immature lung to preserve lung development. Although primarily intended for different purposes, recombinant TRAIL and TRAIL agonists are readily available as a novel therapy to validate its safety in preclinical evaluation that was not within the focus of our studies and to specify therapeutic NFκB signaling stabilizing candidates for clinical trials [39].

Taken together, our data add novel mechanistic insights to the knowledge on cytokine and growth factor signaling in the immature lung and the development of BPD. The presented results categorize TRAIL as a growth factor and not as death inducing ligand or pro-inflammatory cytokine. Our study can serve as basis for the further detailed investigation of the role of TRAIL in lung development and to decipher its action on the different lung cell populations. Lastly, the presented advances can benefit lung diseases of all ages where lung regeneration is a research priority.

## Supplementary information

Supplementary Information

Supplementary Information - Original western blot

aj-checklist

## Data Availability

The datasets generated during and analysed during the current study are available from the corresponding author (Harald.Ehrhardt@paediat.med.uni-giessen.de) on reasonable request.
