# Supplementary figures and images for: TRAIL protects the immature lung from hyperoxic injury
Source: Cell Death Dis. 2022 Jul 15;13(7):614. doi: 10.1038/s41419-022-05072-5 (PMC9287454; doi:10.1038/s41419-022-05072-5)

**Supplementary Information**

Primer list


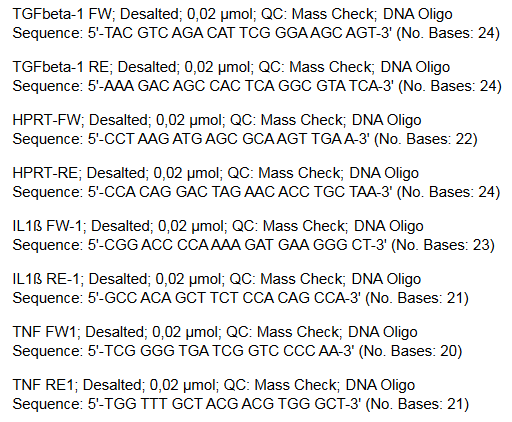

Supplement: Supplementary file 1 — Supplementary Information [file 41419_2022_5072_MOESM1_ESM.docx]

Figure 3G


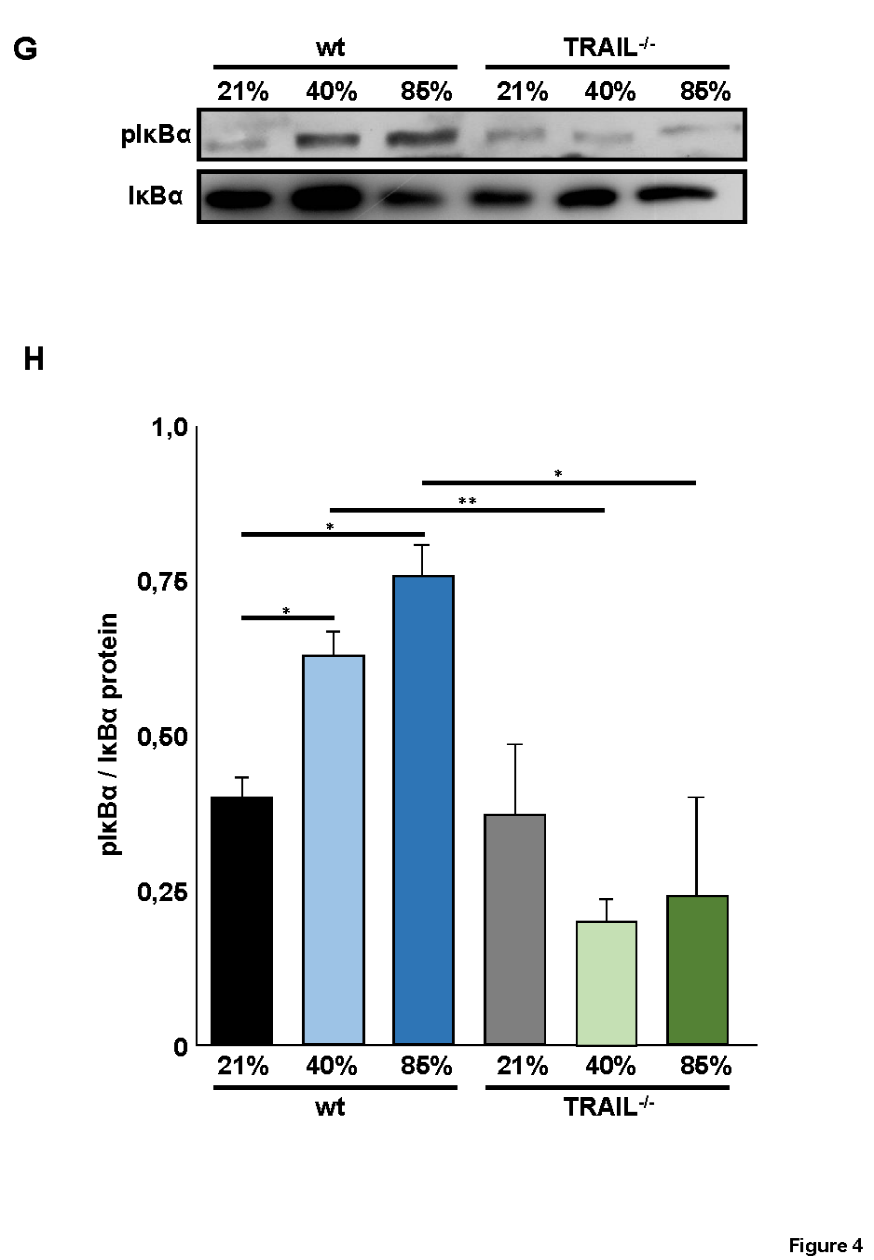


pIĸBα


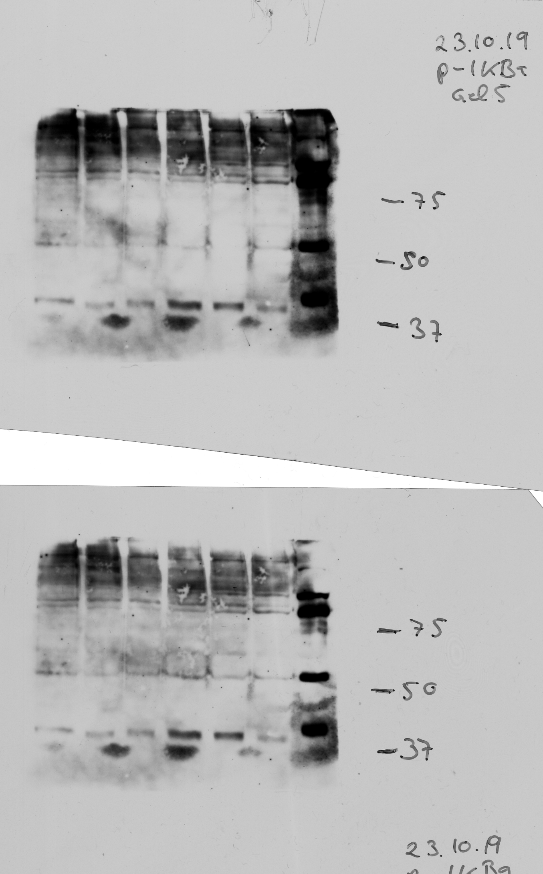


IĸBα


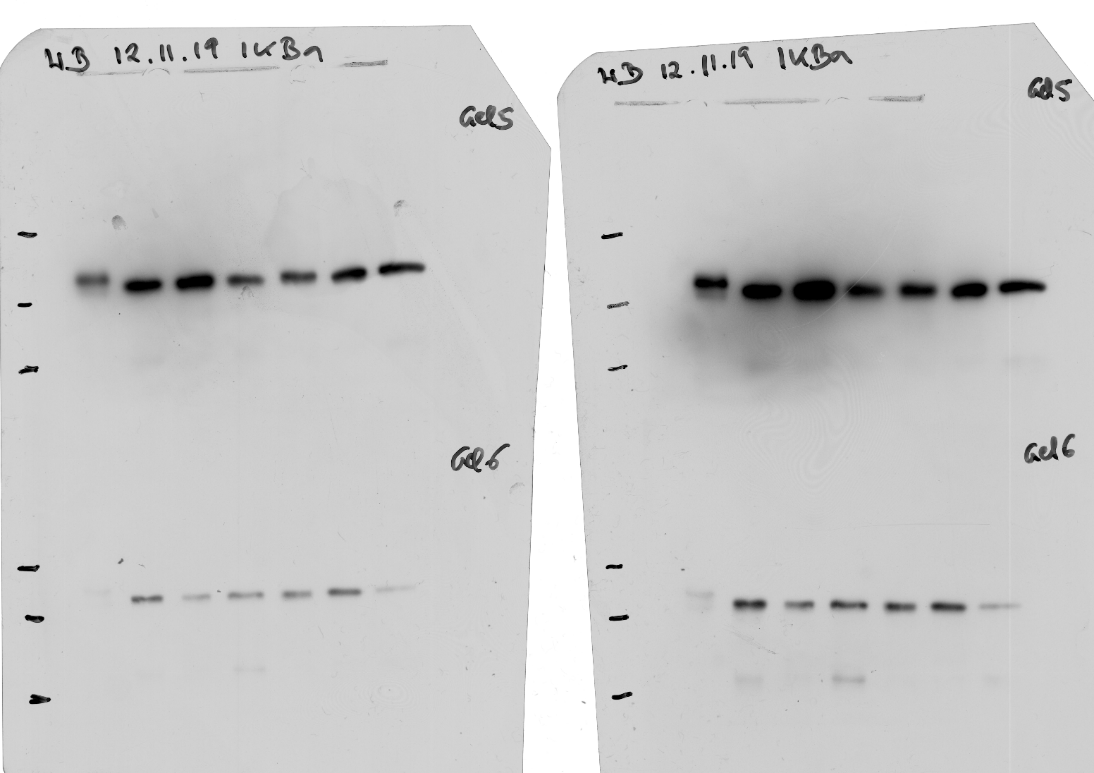

Supplement: Supplementary file 2 — Supplementary Information - Original western blot [file 41419_2022_5072_MOESM2_ESM.docx]
